# ROS attenuates TET2-dependent ZO-1 epigenetic expression in cerebral vascular endothelial cells

**DOI:** 10.1186/s12987-022-00370-8

**Published:** 2022-09-08

**Authors:** Lan Wang, Bei Mao, Keyang Fan, Renqiang Sun, Jialong Zhang, Huazheng Liang, Ying Liu

**Affiliations:** 1 Department of Pathology, School of Basic Medical Science, Fudan University, Yixueyuan 138, Shanghai, China; 2 Molecular and Cell Biology Lab, Institutes of Biomedical Sciences, Shanghai Medical College of Fudan University, Yixueyuan Rd. 138, Shanghai, 200032 China; 3 Translational Research Institute of Brain and Brain-Like Intelligence, Shanghai Fourth People’s Hospital, School of Medicine, Tongji University, 1279 Sanmen Road, Hongkou District, Shanghai, 200434 China; 4 Clinical Research Center for Anesthesiology and Perioperative Medicine, Tongji University, Shanghai, China; 5 Department of Anaesthesiology and Perioperative Medicine, Shanghai Fourth People’s Hospital, School of Medicine, Tongji University, Shanghai, China; 6 Shanghai Key Laboratory of Anesthesiology and Brain Functional Modulation, Shanghai, China

**Keywords:** The ten–eleven translocation methyl cytosine dioxygenase, 5-hydroxymethylcytosine, Aging, Blood brain barrier, Zonula occludens-1, Vascular endothelial cells

## Abstract

**Aims:**

To investigate whether DNA active demethylase TET regulates the expression of tight junction proteins in endothelial cells of the blood–brain barrier (BBB).

**Methods:**

Correlations between TET2 activity (indicated by its catalytic product 5hmC) and the expression of BBB tight junction proteins were examined in Tet2 knockout mice and post-mortem human brain tissues. In cultured endothelial cells, the impact of changes of TET activity on the expression of tight junction protein, ZO-1, was studied. BBB permeability assays were performed in Tet2 knockout mice.

**Results:**

It was found that the level of 5hmC decreased in brain microvascular endothelial cells of aging mice. In Tet2 knockout mice, the level of 5hmC in endothelial cells was weaker and significantly correlated with the reduced expression of tight junction protein ZO-1. In cultured endothelial cells, H_2_O_2_ significantly decreased the expression of 5hmC and ZO-1. Tet2 knock-down using siRNA significantly downregulated the expression of ZO-1 in endothelial cells. hMeChip-PCR showed that H_2_O_2_ decreased the level of 5hmC in the ZO-1 promoter region, which was rescued by N-acetyl cysteine (NAC). Consistently, Tet2 knock-down using siRNA significantly downregulated the level of 5hmC in the ZO-1 promoter region. It was also found that the level of 5hmC decreased in endothelial cells of aging human brains compared with that of adult brains, and the level of ZO-1 was positively correlated with that of 5hmC in microvascular endothelial cells.

**Conclusions:**

These findings suggest that TET activity is essential in regulating ZO-1 expression of BBB. It might be a potential target for neuroprotection during aging and in diverse neurological conditions.

**Supplementary Information:**

The online version contains supplementary material available at 10.1186/s12987-022-00370-8.

## Keypoints

1. The expression of tight junction protein ZO-1 decreased significantly in the aged human brain.
2. TET activity of cerebral endothelial cells declines during aging.
3. TET activity is essential in regulating the expression of ZO-1 by endothelial cells of BBB.

**Supplementary Information:**

The online version contains supplementary material available at 10.1186/s12987-022-00370-8.

## Introduction

Epigenetic regulations are functional chromatin alterations that are not caused by changes in nucleotide sequences [1]. As one of the key epigenetic modifications, mammalian genomic DNA methylation is nearly universal in eukaryotic genomes. Hypomethylation and hypermethylation result in increased or decreased expression of genes, respectively. In mammals, epigenetic modifications of DNA predominantly involve the addition of a methyl group to carbon five of the cytosine base (C), subsequently generating 5-methylcytosine (5mC), which is catalyzed by DNA methyltransferases (DNMTs) [2]. DNA methylation has been considered irreversible until the discovery of the structure and function of the demethylase TET (ten-eleven translocation) protein which renders DNA methylation a dynamic, reversible, and actively regulated process. TET oxidizes 5mC to 5-hydroxymethylcytosine (5hmC) [3]. Sequentially, TET enzymes hydroxylate 5hmC to produce 5-formylcytosine (5fC) and 5-carboxylcytosine (5caC). Thymine DNA Glycosylase (TDG) recognizes the intermediary bases 5fC and 5caC and disrupts the glycosidic bond, leading to an apyrimidinic site. After that, base-excision repair (BER) enzymes subsequently repair the apyrimidinic site and T:G mismatches to generate cytosine [4]. TET belongs to the family of Fe (II)—and α-ketoglutarate (α-KG)-dependent dioxygenases. Different TET isoforms are expressed in various cells and organs. TET1 expression is present in embryonic stem cells, and TET3 is mainly enriched in oocytes, whereas TET2 is widely distributed temporally and spatially [5].

TET activity, as measured by the catalytic product of the TET enzyme, 5hmC, is significantly downregulated across different conditions, such as during the development of stem cells [6], deprivation of oxygen in hypoxic cells [7], 2-hydroxyglutarate (2-HG) treatment which acts as an α-KG antagonist by binding to the same space in the catalytic site and competitively inhibits the activity of TET proteins [8]. All above indicate a potentially fundamental role of 5hmC and cytosine epigenetic modification in various physiological and pathological conditions.

Meisel reported that part of the intestinal barrier of Tet2-KO mice was damaged as evidenced by decreased expression of a tight junction protein—zonula occludens 1 (ZO-1) by jejunal epithelial cells and increased permeability of the intestinal barrier, which results in lactobacillus residing in the small intestine entering the blood and the spleen. Ectopic growth of lactobacillus induces the expression of interleukin-6 (IL-6) which stimulates myeloid cell proliferation and subsequent preleukemic myeloproliferation [9].

The blood–brain barrier (BBB) is another critical barrier of the human body, whose structure is very similar to that of the intestine, such as the tight junction proteins of claudins and ZO-1 [10]. These tight junction proteins, along with the immune barrier composed of phagocytes and other inflammatory cells, form a potential physical barrier confining the transgression of pathogens, diffusion of solutes in the blood, and the entry of macromolecules or hydrophilic molecules. This highly selective semi-permeable barrier maintains the homeostasis of the internal environment [11]. Based on the similarity between these two barriers, we speculated that inhibition of TET2 activity of cerebrovascular endothelial cells might decrease the expression of ZO-1 in the brain, leading to the structural damage of BBB.

The accumulation of oxidative stress participants in aging induces BBB damage [12]. Oxidative stress has been reported to influence TET hydroxylase activity [13]. It is, therefore, reasonable to hypothesize that oxidative stress during aging induces the decrease in TET2 activities of cerebrovascular endothelial cells, leading to decreased expression of tight junction proteins, BBB destruction, and the potential occurrence of AD. The present study aimed to investigate the developmental changes of 5hmC and ZO-1 expression of endothelial cells in aged human brains and to explore evidence of direct regulation of ZO-1 expression by TET2 in Tet2-knockout mice and cultured endothelial cells.

## Methods

### Antibodies and reagents

Antibodies against ZO-1 (Abcam, ab190085; dilution 1:1,000), 5hmC (Active Motif, #39769, dilution 1:1,000), GFAP (Abcam, ab7260, dilution 1:1000), NeuN (Abcam, ab177487, dilution 1:1000), CD68 (Abcam, ab237988, 1:1000) and Claudin-5 (Abcam, ab131259, dilution 1:500) were used for immunohistochemistry staining. Antibody against 5hmC (Active Motif, #39769, dilution 1:5,000) was used for dot-blotting. Antibody against ZO-1 (Proteintech, 22601-1-AP, dilution 1:1000) and TET2 (Proteintech, 21207-1-AP, dilution 1:1000) were used for western-blotting.

Hydrogen peroxide solution (H_2_O_2_) (Sigma, 323381), N-acetyl cysteine (NAC) (Sigma, A7250), and DAPI (Sigma, D9542) were commercially obtained.

Fluorescein isothiocyanate (FITC)-conjugated dextran (40 kDa) and FITC-conjugated dextran 10 kDa were purchased from Thermo Fisher Scientific Inc. (Waltham, MA USA).

### Cell culture and treatment

Human umbilical vein endothelial cells (HUVECs), human brain microvascular endothelial cells (HBMECs), and immortalised mouse brain endothelial bEnd.3 cells were obtained from the American Type Culture Collection (ATCC). HUVECs, HBMECs, and bEnd.3 cells were grown in the endothelial cell medium (ECM) (Cat. No. 1001, ScienCell, USA) containing 5% FBS (Cat. No. 0025, ScienCell, USA), 1% endothelial cell growth supplement (ECGS, ScienCell, US), and 1% antibiotic solution (P/S, ScienCell, USA). Following 12-h serum deprivation synchronization, HUVECs and bEnd.3 cells were treated with H_2_O_2_ and NAC supplement. Cells were plated in complete culture media with 10 μM H_2_O_2_ for 6 h, then exchanged the medium supplemented with 1 mM NAC for another 6 h.

For TET2 siRNA transfection, TET2 siRNAs were commercially acquired from Hanbio Ltd, Shanghai, China (sequences were list in Table 2). HUVECs and HBMECs were seeded in 6-well plates and transfected with control siRNA or siRNA for TET2 using riboFECT™ CP Transfection Kit (Hanbio Ltd, Shanghai, China) according to the manufacturer’s instructions after 70–80% cell confluence was reached. The final concentration of siRNA solution was 20 μM. MISSION^®^ siRNA Universal Negative Control (Sigma-Aldrich) was used as control.

### Animals

Tet2-floxed mice and EIIa-Cre mice were kindly provided by Dr. Ye Dan (Fudan University), which were described previously [14]. Quantitative PCR confirmed that the Tet2 mRNA level was reduced by 90% or more in the tail tissue isolated from Tet2-KO mice. Littermate control mice were used as controls. These mice were housed in the laboratory animal centre of Fudan University. All experiments were approved by the Laboratory Animal Management and Ethics Committee of Fudan University by complying with the Chinese Guidelines on the Care and Use of Laboratory Animals (20180302-051).

### Human brain tissue

Post-mortem human archival brain tissue was collected by the Department of Pathology, School of Basic Medical Sciences, Fudan University. All procedures associated with the acquisition and usage of the human brain samples were approved by the Ethics Committee of Fudan University (2019-Y004). These procedures were conducted by complying with the Helsinki Declaration (1964, amended most recently in 2013) [15]. Clinical histories and autopsy records were reviewed, and samples from patients with cardiovascular disorders were excluded. Brain samples with known hypoxic damage were excluded. Brain samples were evaluated by two neuropathologists as no microscopic abnormality, except brains of old people with mild age-related changes. The average post-mortem interval was 40.13 ± 15.59 h. Formalin-fixed and paraffin-embedded tissue was obtained from the temporal cortex (Brodmann area 21/22). Eight cases were in the adult group with their age less than 60, and 8 cases in the aged group. Case details were provided in Table 1.

**Table 1:** Demographics of Human brain donors

Group	Age	Gender	Cause of death	Post mortem interval (h)	Neuropathology
Aged	66	Male	Lobular pneumonia	40	None
	98	Female	Lobular pneumonia	36	None
	66	Male	Pulmonary thromboembolism	72	None
	90	Female	Multiple organ dysfunction syndrome	36	None
	83	Male	Lobular pneumonia	48	None
	88	Female	Cachexia	24	None
	74	Female	Lobular pneumonia	32	Mild edema
	89	Female	Cachexia	36	none
Adult	29	Female	Septic shock	56	Mild edema
	28	Male	Acute complete intestinal obstruction	70	Mild edema
	40	Female	pulmonary thromboembolism	50	None
	52	Male	Lobular pneumonia	38	None
	54	Female	Respiratory tract obstruction	20	None
	52	Male	Heart failure	36	None
	54	Female	Asphyxia	24	None
	58	Female	Cachexia	24	None

### 5hmC dot-blotting

The procedure for the dot-blotting assay was modified from the procedure described previously [13]. Briefly, genomic DNA was spotted on a nitrocellulose membrane (Whatman), which was placed under an ultraviolet lamp for 20 min to crosslink the DNA. Subsequently, the membrane was blocked with 5% milk in TBST for 1 h, followed by the incubation with the primary anti-5hmC antibody at 4 °C overnight. After incubation with horseradish peroxidase-conjugated anti-rabbit IgG (GenScript) for 1 h at room temperature, the membrane was washed with TBST three times and then scanned with a Typhoon scanner (GE Healthcare).

### Immunofluorescence and microscopy

Cells were plated at 1 × 10^6^ per well on glass coverslips in 6-well culture plates and stimulated as described above. Cells were incubated overnight at 4 °C with a rabbit anti-ZO-1 antibody (1:1000; Chemicon). The primary antibody was detected with an isotype-specific anti-rabbit secondary antibody (1:1000) conjugated with Alexa 488 (Invitrogen). Negative control samples were performed without the primary antibody. 4′,6-Diamidino-2-phenylindole (DAPI, Invitrogen) was used for counterstaining.

Fluorescent images were captured under an LSM 510META confocal microscope (Carl Zeiss).

### Western blot analysis

For western blot analysis, proteins were extracted by the radioimmunoprecipitation assay buffer (RIPA) containing complete protease and phosphatase inhibitor cocktails (Selleck, Houston, TX, USA). Protein concentrations were determined using the BCA Protein Assay Kit (Thermo Fisher Scientific, Waltham, MA, USA). The blots were incubated with primary antibodies followed by the incubation with HRP-conjugated secondary antibodies (Abcam, Cambridge, UK). The protein–antibody complexes were visualised using an enhanced chemiluminescence kit (Thermo Fisher Scientific, Waltham, MA, USA).

### hMeDIP-qPCR analyses

Genomic DNA from cells was prepared using a DNA isolation kit (QIAGEN). The hMeDIP assay was performed as previously described [16]. Briefly, genomic DNA was sonicated to 300–500 bp fragments, denatured, then immunoprecipitated with an anti-5hmC antibody (Active Motif) or IgG control antibody (Active Motif) and protein A magnetic Dynabeads (Millipore). After washing three times, beads were treated with proteinase K for 3 h. Chip’d DNA was purified with a QIAquick PCR purification kit (Qiagen) by following the instructions from the manufacturer. 2 μg of each genomic DNA was used to run quantitative Real-time PCR (qPCR). qPCR amplification was performed using the SYBR Green system (Takara, Dalian, China) in an Applied Biosystems thermal cycler with StepOnePlus™ RT‐PCR System (ThermoFisher, MA, USA). Relative quantification of target genes was conducted using the 2 − ΔΔCT method. Isotype-specific IgG was used as a control. All primer sequences for qRT-PCR were listed in Table 2.

**Table 2:** primers of Tet 2 siRNA and hMeDIP-qPCR

Tet2 siRNA-1	F: 5′-CCCAGAGUCCUAAUCCAUCUATT-3′ R: 5′-UAGAUGGAUUAGGACUCUGGGTT-3′
Tet2 siRNA-2	F: 5′-AAGCUAGCGUCUGGUGAAGAATT-3′ R: 5′-UUCUUCACCAGACGCCUAGCUUTT-3′
Tet2 siRNA-3	F: 5′-CAAGGCAGUGCUAAUGCGUAATT-3′ R: 5′-UUAGGCAUUAGCACUGCCUUGTT-3′
SiRNA NC	F: 5′-UUCUCCGAACGUGUCACGUTT-3′ R: 5′-ACGUGACACGUUCGGAGAATT-3′
Homo Tjp1-CpG1- Homo Tjp1-CpG-	F: 5′-ATAAGTAATACAGCATGAAA-3′ R: 5′-TGCTGGCCGGGAGCTGCTGT-3′
Homo Tjp1-CpG2	F: 5′-AGCTATGCACCTGCCCAGTA-3′ R: 5′-TCCCTCGCTGGCGGCTGTCT-3′
Homo Tjp1-CpG3	F: 5′-CAAATAAACATCTCCCGAGA-3′ R: 5′-AGTGGGTGGCCGCGCTCGGA-3′
Homo Tjp1-CpG4	F: 5′-CCTAGAGACCTGTGACATCT-3′ R: 5′-AACAAGAATTTCAGCAATCTT-3′

### Immunohistochemistry staining (IHC)

For IHC analysis, the labelled streptavidin–biotin (LSAB) method was applied using commercial kits (Dako Corporation, Santa Barbara, CA, USA). Briefly, paraffin embedded sections were deparaffinised and rehydrated following the standard protocol. The sections were then transferred to the antigen retrieval solution (Tris–EDTA pH: 8.0) for incubation for 10 min, followed by incubation with 3% H_2_O_2_ to eliminate the endogenous peroxidase activity, and blocked with 5% normal goat serum. After treatment with 2 N HCl for 15 min at room temperature, sections were neutralised with 100 mM Tris–HCl (pH 8.5) for 10 min and then washed three times with PBS, followed by incubation with primary anti-5hmC antibody at 37 °C for 1 h. Horseradish peroxidase (HRP)-conjugated secondary antibody (Dako Corporation) was then applied and incubated at 37 °C for 1 h. For ZO-1, CD68, NeuN, and GFAP IHC staining, the LSAB method was used as described above, omitting the treatment with 2 N HCl and the ensuing Tris–HCl neutralization.

To semi-quantify the IHC staining of ZO-1, GFAP, CD68, and NeuN, five fields (173 µm^2^ each) from each sample were randomly selected and microscopically examined. Images were captured using a charge-coupled device (CCD) camera and analyzed using the IMT i-Solution software (IMT i-Solution, Inc., Burnaby, BC, Canada) to get the staining intensity. All IHC image analysis values were calculated as mean ± SEM.

To quantify the endothelial 5hmC intensity, immunoreaction scores (IRS) of 5hmC on endothelial cells were calculated based on the staining intensity (SP) and the positive staining percentage (SI) of the cells. SI was scored as follows: 0: 0–1%; 1: 2–25%; 2: 26–50%; 3: 51–75%; and 4: 75–100%. SP was subjectively scored as follows: 0, no staining; 1, weak but definite staining; 2, moderate staining; and 3, intense staining. The IRS was calculated as IRS = SP × SI. The total score was 12, and the range was from 0 to 12.

### Measurement of the permeability of the endothelial monolayer

To evaluate the paracellular permeability, HUVECs and HBMECs cells were seeded in the upper chamber of 12-well Transwell inserts (3460, CORNING, New York, NY, USA) to form a confluent monolayer. After washing these cells with the blank medium, FITC-Dextran (1 mg/ml, Sigma, St. Louis, MO, USA) was added to the upper chamber. After incubating at 37 °C for 6 h, the fluorescence intensity of FITC-Dextran transgressed to the lower chamber was measured by using a multimode microplate reader (Synergy H1, Biotek, Inc., Suite F Layton, UT, USA) at the excitation wavelength of 485 nm and emission at 530 nm. The permeability coefficient was calculated using the following equation: Pdextran = (RFUlower/RFUupper) (V) (1/time) (1/area) and normalized to control untreated cultures.

### FITC-dextran extravasation

Dextran of variable sizes coupled with FITC was used to study BBB permeability. Mice received 20 mg/ml of FITC-dextran (10 kDa or 40 kDa) dissolved in isotonic saline at a dose of 0.4 ml/kg. Five minutes after injection, mice were anesthetized with isoflurane and sacrificed. The brains were isolated and fixed with 4% PFA for 6 h, then incubated in 10% glycerol for 18–24 h at 4 °C. After being embedded in OCT and snap frozen, brains were cut into 40 μm thick coronal sections. Fluorescent images were captured under an LSM 510 META confocal microscope (Carl Zeiss, Thornwood, NY, USA).

### Statistical analysis

GraphPad Prism 8 was used to assess the significance of all data. One-way ANOVA followed by the post-hoc test (e.g., Dunnett’s Test) was used to assess parameters between multiple groups, and an unpaired two-tailed Student’s t-test for comparisons between two groups. Statistical significance was set at p < 0.05.

## Results

### Changes of 5hmC, ZO-1, and CD11b in Tet2-/- mice

**Fig. 1:**
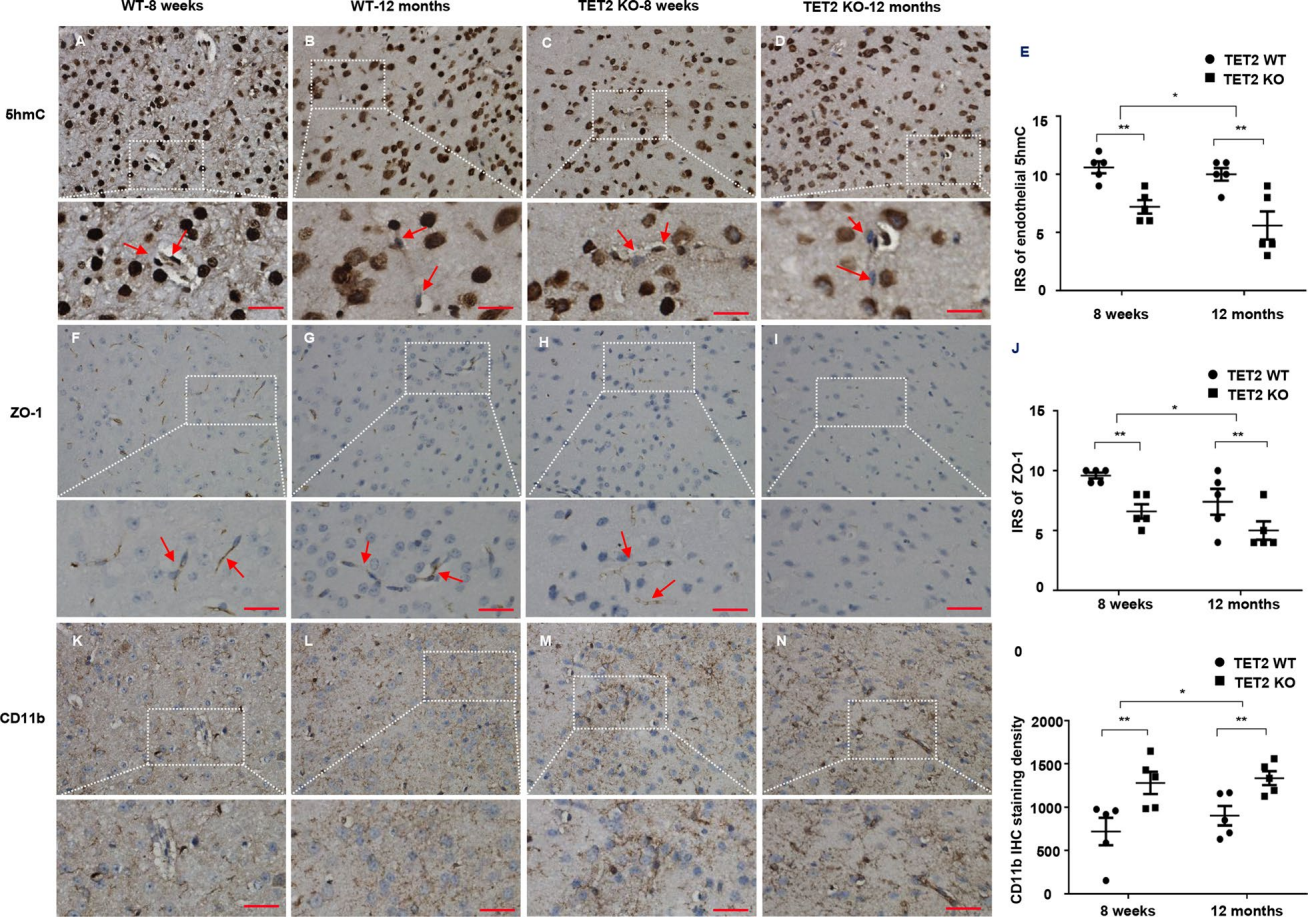
Changes of 5hmC, ZO-1, and CD11b in Tet2-/- mice. **A–D** 5hmC signal in endothelial cells (arrow) of 8-week and 12-month old wild type and Tet2 KO mice. **E** Semi-quantitative analysis of 5hmC staining of brain endothelial cells in wild type and Tet2 KO mice. **F–I** ZO-1 expression by endothelial cells (arrow) of 8-week and 12-month old wild type and Tet2 KO mice. **J** Semi-quantitative analysis of ZO-1 expression by endothelial cells. **K–N**. CD11b signal in endothelial cells (arrow) of 8-week and 12-month old wild type and Tet2 KO mice. **O** Semi-quantitative analysis of CD11b expression by endothelial cells. Scale bars: 50 μm. All data were shown as the mean ± SEM. The p values were determined by the two-tailed t-test. Values of p < 0.05 were considered statistically significant. * and ** denoted p < 0.05 and p < 0.01, respectively

Meisel et al. reported that part of the intestinal barrier of Tet2-KO mice was damaged [9], but there is no report on the impact of Tet2 knockout on ZO-1 expression in the brain endothelial cells. We first detected the expression of ZO-1 in brain endothelial cells of Tet2-KO mice. In the brain of 8-week-old wild-type mice, 5hmC was strongly positive in endothelial cells (Fig. 1A). Patches of 5hmC signal were lost in endothelial cells of 12-month old mice (Fig. 1C). 5hmC signal was weaker in endothelial cells in both 8-week-old (Fig. 1B) and 12-month-old Tet2 KO mice (Fig. 1D). Semi-quantitative analysis of 5hmC in endothelial cells was shown in Fig. 1E. Compared with wild-type mice of the same age, the expression of 5hmC in endothelial cells decreased significantly in Tet2-KO mice. Like wild-type mice, the loss of 5hmC in endothelial cells in 12-month-old Tet2 KO mice was more prominent than that of wild-type mice compared with their 8-week-old counterparts. These indicate that aging modulates the endogenous TET2 activity.

A similar phenomenon was observed in ZO-1 expression by endothelial cells (Fig. 1F–J). ZO-1 positive staining was continuous in endothelial cells of wild-type mice (Fig. 1F, H), which was significantly reduced in Tet2-KO mice (Fig. 1G, I). Semi-quantitative analysis was shown in Fig. 1J. There is no difference in Claudin-5, another tight junction protein of endothelial cells (Additional file 1: Fig. S1). This indicates that Tet2 knockout specifically regulates the expression of ZO-1 protein.

Microglia is generally activated after BBB impairment. In our study, it was found that the density of CD 11b positive signal in Tet2-KO mice was significantly increased in regions surrounding blood vessels (Fig. 1K, M) compared with that of wild-type mice (Fig. 1L, N) at both 8 weeks and 12 months of age. Semi-quantitative analysis was shown in Fig. 1O.

### TET2 activity regulates the expression of ZO-1

**Fig. 2:**
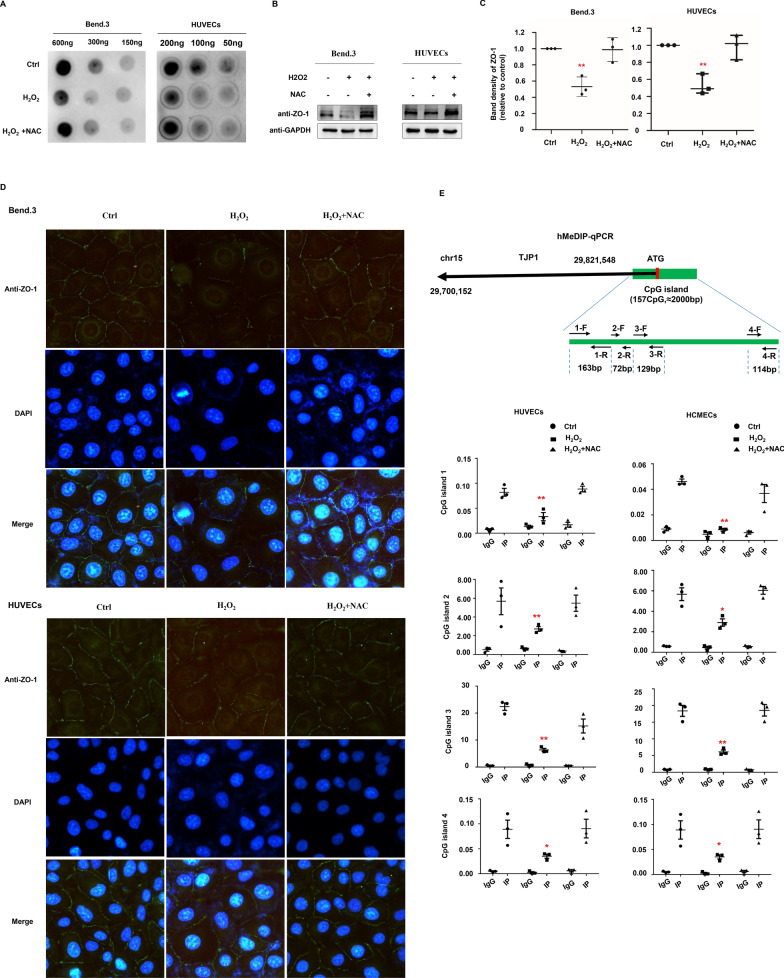
ROS epigenetically compromised ZO-1 expression. **A** Bend.3 and HUVECs were treated with or without 10 μM H_2_O_2_ for 6 h and supplemented with or without 1 mM NAC for an extended 6 h for blotting 5hmC as indicated. **B** The expression of ZO-1 in Bend.3 and HUVECs treated with or without 10 μM H_2_O_2_ for 6 h and supplemented with or without 1 mM NAC for an extended 6 h. **C** Semi-quantitative analysis of ZO-1 positive bands of endothelial cells. **D** Immunofluorescence staining of ZO-1 expression in endothelial cells treated with H_2_O_2_ and supplemented with NAC as indicated. **E** HUVECs and HCMECs treated with H_2_O_2_ then supplement with NAC. Scale bars: 50 μm. All data were shown as the mean ± SEM. The p values were determined by the two-tailed t-test. Values of p < 0.05 were considered statistically significant. *and ** denoted p < 0.05 and p < 0.01, respectively

It is known that aging results in mitochondrial malfunction and reduced energy supply to diverse organelles. Consequently, cellular reactive oxidative stress (ROS) increases in endothelial cells [17]. The activity of TET2 displayed an absolute requirement for reduced ferrous iron (Fe2+) [18]. ROS can reduce TET activity through the inactivation of Fe^2+^ in its catalytic domain. As expected, H_2_O_2_ treatment significantly decreased the expression of 5hmC in both Bend.3 and HUVECs, whereas co-treatment with N-acetyl cysteine (NAC) significantly reversed the decrease of 5hmC by H_2_O_2_ (Fig. 2A). Western blot showed ZO-1 expression was also downregulated by the addition of H_2_O_2_ and rescued by co-treatment with NAC (Fig. 2B, C). Immunofluorescence staining showed that after H_2_O_2_ treatment, the linear expression of ZO-1 protein on the endothelial cell membrane was discontinuous or disappeared. After NAC treatment, ZO-1 expression was partially rescued (Fig. 2D). DNA methylation at CpG dinucleotides of the promoter region is a hallmark of silenced gene expression. Higher 5hmC levels usually indicate lower methylation and increased expression of corresponding genes. MeChip-PCR was performed to detect the methylation level of CpG islands of the ZO-1 promoter. In this test, 5hmC modulated DNA was pulled-down first, and then quantified using PCR with the assistance of four pairs of primers targeting the ZO-1 promoter CpG islands. It was found that the level of 5hmC in the ZO-1 promoter region was significantly reduced by H_2_O_2_, whereas NAC significantly attenuated this decrease of 5hmC (Fig. 2E).

### TET2 silencing selectively inhibited the expression of ZO-1 and increased endothelial permeability

**Fig. 3:**
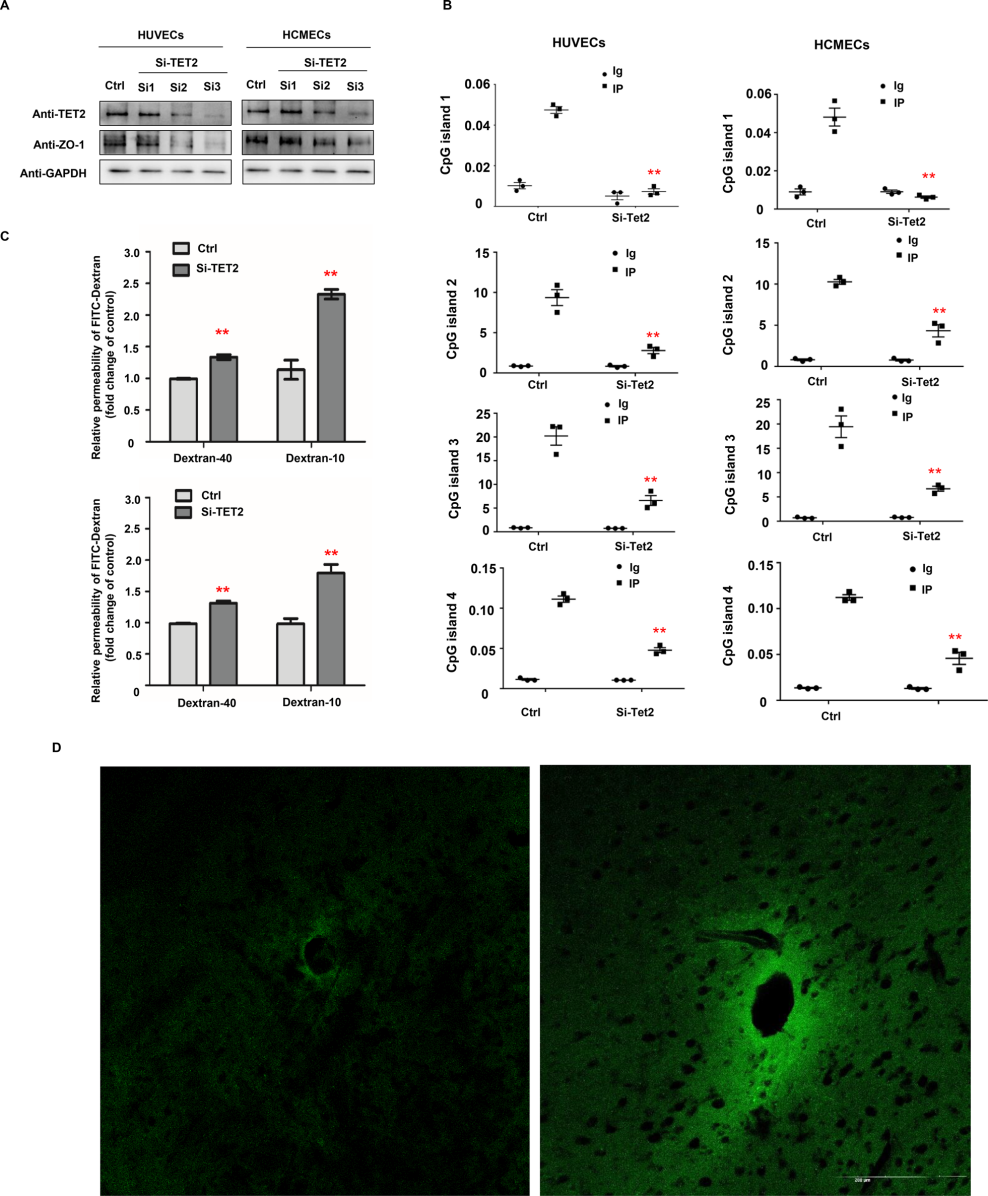
TET2 silencing selectively inhibited the expression of ZO-1 and increased endothelial permeability. **A** The expression of ZO-1 in endothelial cells was decreased after Tet2 was knocked down by siRNA. **B** hMeChip-PCR detected the 5hmC modification of CpG dinucleotides of the promoter region of ZO-1 in endothelial cell before and after knocking down TET. **C** Quantitative data analysis of the permeability of the endothelial monolayer with the assistance of FITC-dextran. **D**, **E** Representative images of FITC-dextran extravasation in the mouse brain. In the brain of wild-type mice, very weak fluorescence appeared in the brain parenchyma (**D**). In the brain of Tet2 KO mice, the brain parenchyma was filled with bright fluorescent FITC-dextran (10 kDa) on a bright green background indicative of extravasation of FITC-dextran across the BBB (**E**). Scale bars: 100 μm. All data were shown as the mean ± SEM. The p values were determined by the two-tailed t-test. Values of p < 0.05 were considered statistically significant. *and ** denoted p < 0.05 and p < 0.01, respectively

To test whether the activity of TET2 directly regulates the expression of ZO-1, we knocked down TET2 expression by siRNAs. It was found that the expression of ZO-1 in endothelial cells correlated with the level of Tet2 (Fig. 3A). There is no difference in Claudin-5 expression in endothelial cells after H_2_O_2_ treatment (Additional file 1: Fig. S2). Consistent with H_2_O_2_ treatment, the 5hmC level at the ZO-1 promoter region was significantly reduced after Tet2 knockdown in endothelial cells (Fig. 3B).

Next, we investigated the importance of TET2 expression in protecting cerebral microvascular integrity. We found that the paracellular diffusion of FITC-dextran across the endothelial monolayer increased in the TET2 siRNA group. The fluorescence intensity of 10 kDa-FITC-dextran extravagated into the lower chamber in Tet2 knockdown groups was 1.83 ± 0.08 times that of the control group, whereas 40 kDa-FITC-dextran extravagated into the lower chamber in Tet2 knockdown groups was 1.33 ± 0.047 times that of the control group.

We evaluated the impact of TET2 inhibition on BBB permeability in vivo. 10 kDa-FITC-dextran was applied as a tracer in morphological studies of vascular permeability. In wild-type mice, there is no fluorescence in the interstitial space (Fig. 3C). In contrast, Tet2-KO mice showed diffuse fluorescence in the interstitial space (Fig. 3D).

Taken together, our findings suggest that TET2 activity is essential in regulating cerebral endothelial permeability.

### 5hmC is markedly reduced in endothelial cells of aged human brains and positively correlated with the expression of ZO-1

**Fig. 4:**
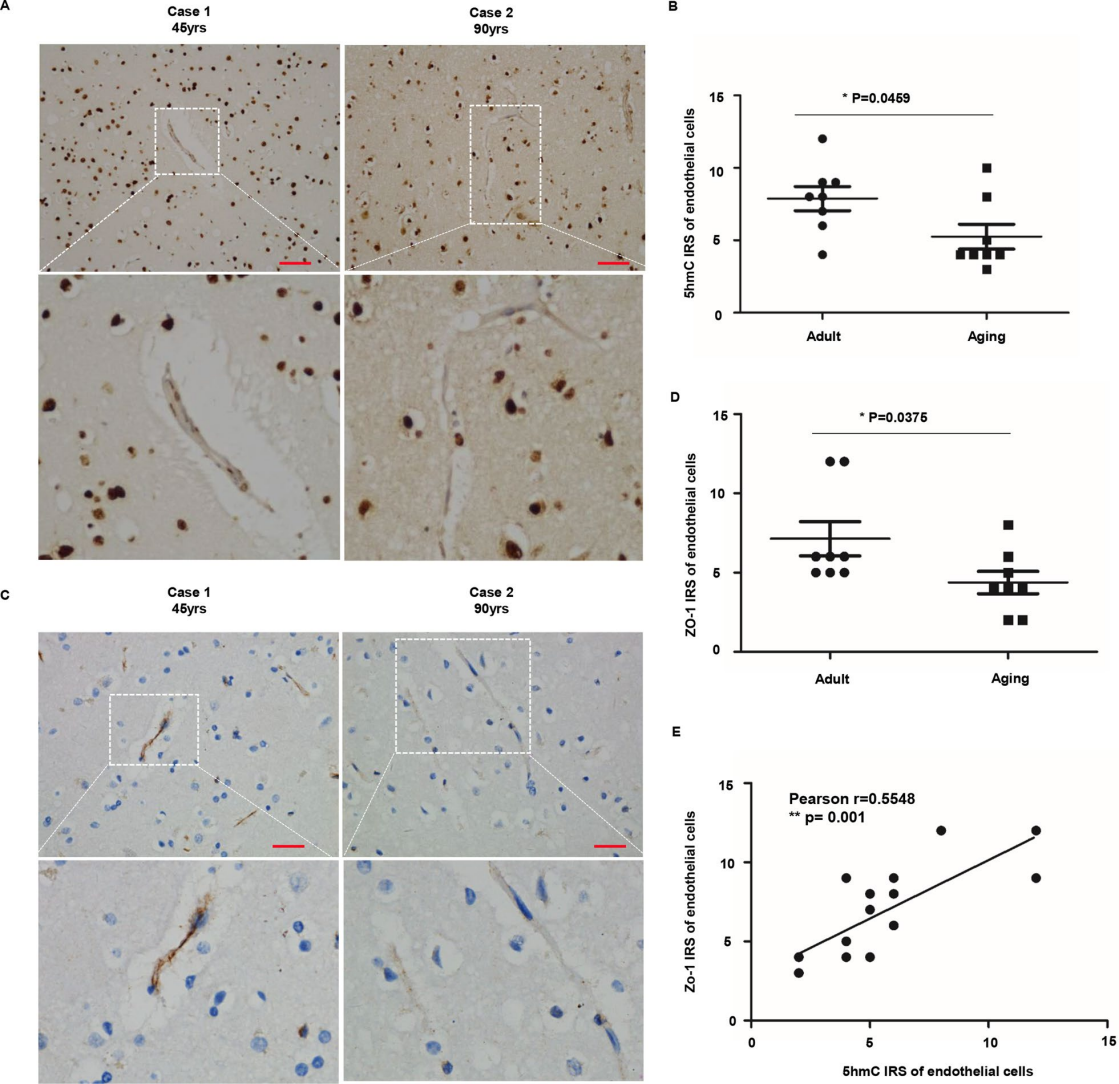
5hmC is markedly reduced in endothelial cells of aged human brains and it is positively correlated with the expression of ZO-1. **A** IHC staining of 5hmC in endothelial cells of adult and aged brains. Insets show the higher magnification of selected regions. **B** Semi-quantitative analysis of 5hmC positive staining in endothelial cells of adult and aged brains. Scale bars: 50 μm. Data were shown as the mean ± SEM. The p values were determined by the two-tailed t-test. Values of p < 0.05 were considered statistically significant. * denoted p = 0.0459. **C** IHC staining of ZO-1 in endothelial cells of adult and aged brains. Insets show the higher magnification of selected regions Scale bars: 50 μm. **D** Semi-quantitative analysis of 5hmC positive staining in endothelial cells of adult and aged brains. Scale bars: 50 μm. Data were shown as the mean ± SEM. The p values were determined by the two-tailed t-test. Values of p < 0.05 were considered statistically significant. * denoted p = 0.0375. **E** The correlation between 5hmC IRS and ZO-1 IRS in endothelial cells of human brains (Pearson correlation γ = 0.5518, **p = 0.001)

IHC against 5hmC was conducted using post-mortem human brains to verify whether 5hmC levels in endothelial cells were altered in the human brain at different ages. We found that 5hmC was positive in endothelial cells of adult brains. Its expression dramatically decreased in endothelial cells of aged brains (Fig. 4A, B). The expression of ZO-1 was also weaker in aged brains compared with that of adults (Fig. 4C, D). This indicates the endothelial tight-junction weakens as age increases. Consistently, 5hmC IRS appeared to be closely associated with ZO-1 IRS in endothelial cells (R = 0.5518, P = 0.0011, Fig. 4E), suggesting the correlation between the expression of ZO-1 and the 5hmC level.

### Simultaneous changes in the human brain

**Fig. 5:**
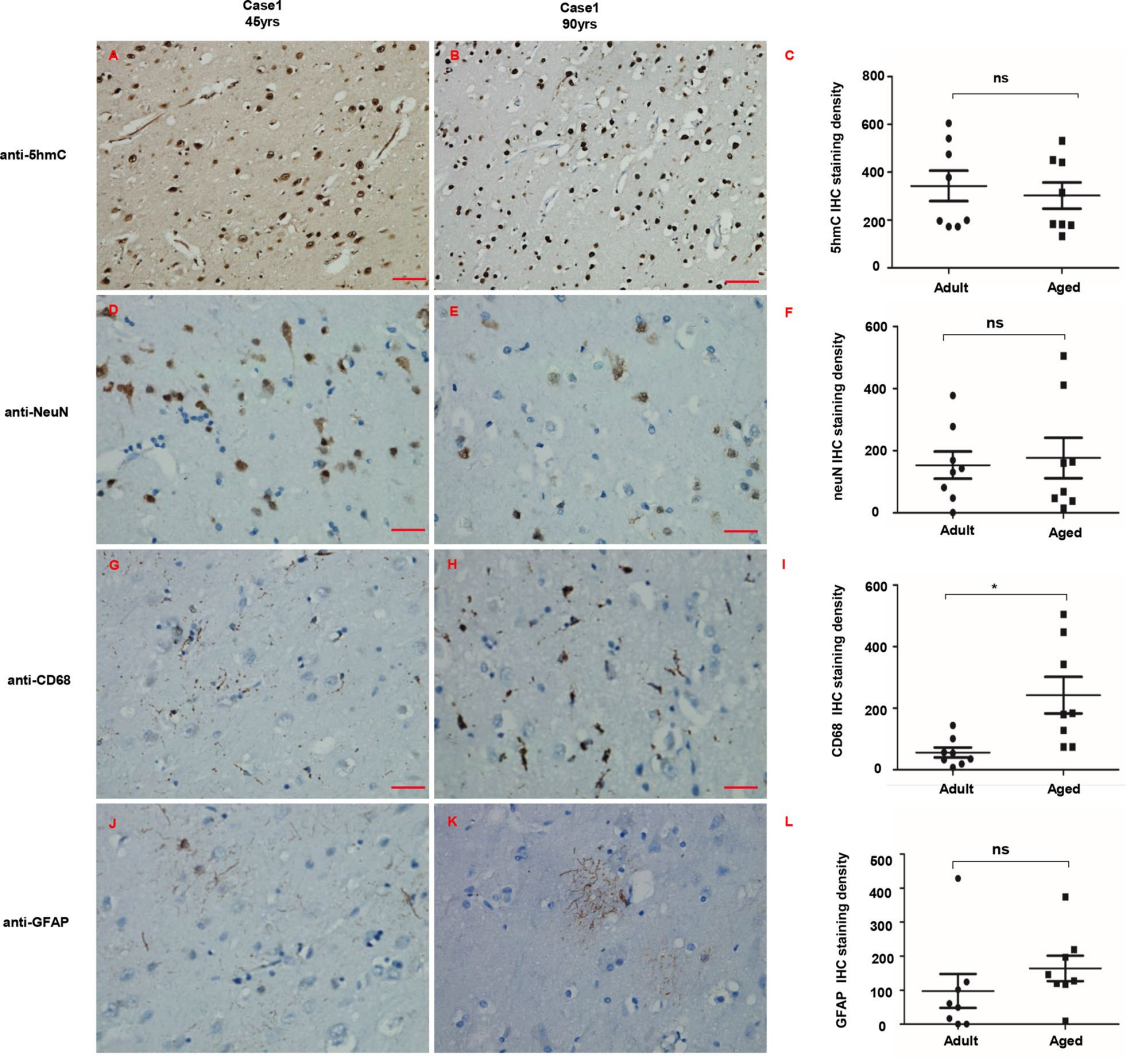
Simultaneous changes in the human brain. **A**, **B** Representative images of the total level of 5hmC in the adult group (**A**) and the aged group (**B**). **C** Semi-quantitative analysis of 5hmC staining. **D**, **E** The number of NeuN positive cells/field in the adult group (**D**) and the aged group (**E**). **F** Semi-quantitative analysis of NeuN staining. **G**, **H.** The number of CD11b positive cells/field in the adult group (**G**) and the aged group (**H**). **I** Semi-quantitative analysis of CD11b staining. **J**, **K** The number of GFAP positive cells/field in the adult group (**J**) and the aged group (**K**). **L** Semi-quantitative analysis of GFAP staining. Scale bars: 50 μm. All data were shown as the mean ± SEM. The p values were determined by the two-tailed t-test. Values of p < 0.05 were considered statistically significant. * denoted p < 0.05; *ns* not significant

The blood–brain barrier (BBB) plays a vital role in maintaining the specialized microenvironment of the neural tissue. It separates the peripheral circulatory system from the brain parenchyma while facilitating communication. Alterations in distinct physiological properties of the BBB lead to BBB breakdown associated with normal aging and various neurodegenerative diseases [19]. Here we examined BBB-associated cells in both adult and aged brains. The total level of 5hmC showed no significant change between the adult and the aged groups (Fig. 5A–C). The number of NeuN positive cells/field seemed to decrease in aged brains, but this change was statistically insignificant (Fig. 5D–F). CD68 positive microglia (Fig. 5G, H) was increased in the aged group, and GFAP positive astrocytes seemed to increase in the aged group but had no statistical significance (Fig. 5J–L). Similarly, claudin-5 was not significantly different between adult and aged brains (Additional file 1: Fig. S3).

## Discussion

The present study examined the impact of Tet2 activity on the expression of ZO-1, a BBB tight junction protein, and the underlying mechanism. We found that the level of 5hmC decreased in brain microvascular endothelial cells of aged mice and it was even weaker in Tet2 knockout mice. Similar decrease was observed in the expression of ZO-1. In cultured endothelial cells, Tet2 knock-down significantly downregulated the expression of ZO-1 in endothelial cells accompanied by decreased levels of 5hmC in the ZO-1 promoter region, suggesting the causal relationship between Tet2 activity and ZO-1 expression. Findings from post-mortem human brains support the findings from cultured endothelial cells and Tet2 knockout mice.

Meisel et al. reported that the intestinal barrier of the jejunum of Tet2-KO mice was damaged, and they found no changes in ZO-1 in epithelial cells of the colon [9]. There are three isotypes of TET in mammalian cells. The distribution of three TET enzymes is related to the development stage and cell types in the mammalian body. TET1 is mainly enriched in embryonic stem cells, TET3 in oocytes, and TET2 is widely distributed spatially and temporally [5]. Here, we found that TET2 activity modified the expression of ZO-1 in cerebral endothelial cells. ZO-1 serves as an important linker molecule of tight junctions which comprise the main component of mechanical barriers in the human body. Whether TET2 dependent ZO-1 expression modulates the permeability of other mechanical barriers such as blood-testis-barrier still needs further verification.

TET activity is suppressed under a variety of pathophysiological conditions. Various metabolites, such as 2-hydroxyglutarate (2-HG), α- KG antagonist, competitively inhibit the activity of TET enzymes in IDH mutant glioma cells [8], and itaconic acid (ITA) inhibits Tet activity in macrophages after LPS treatment [20]. TET enzymes are also inactivated under hypoxia [7] or by mutations in about 15% of myeloid cancers [21]. The decrease of TET activity will decrease the level of 5hmC, and this epigenetic modification of cytosine has a potential role in various physiological and pathological conditions. Previous studies on TET2 in the nervous system often focused on the relationship between the 5hmC level of neurons and their function. Using antibody-based 5hmc immunoprecipitation combined with deep sequencing (hMeDIP SEQ), Gontier identified 345 differential 5-hydroxymethylated regions (DHMRS) in the hippocampus of adult and elderly mice. All DHMRS showed that 5hmC in the hippocampus was partially lost with age and confirmed that TET2 activity was related to the methylation modification of the brain during aging [22]. However, another team found that TET1, TET2, and 5hmC levels in the hippocampus of aged mice were lower than those of adult mice, and there was a positive correlation trend between TET2 and 5hmC, but this decrease was not significant [23]. In our study, we also found that no changes of 5hmC were observed in aged human brain parenchymal cells. Even in Tet2-KO mice, there were no changes of 5hmC in neurons. Since TET proteins have three isoforms and neurons remain relatively static, that may explain the insignificant change in 5hmC of neurons to certain extents.

Tight junction proteins between brain endothelial cells greatly restrict paracellular transport. Therefore, the expression and function of the tight junction proteins are used as a marker of BBB integrity [24]. ZO-1, one of the tight-junction proteins, interact with other tight junction proteins to anchor and tether them to the actin cytoskeleton [25]. Alterations and breakdown of the BBB can occur naturally with aging even in the absence of concomitant conditions that cause cognitive decline and dementia [26]. During healthy aging, there is an age-dependent loss of BBB integrity. BBB integrity can become compromised at the beginning of the loss of tight-junction proteins of the endothelial cell disrupted by excessive oxidative stress accumulated over time. Here, we found that the expression of tight junction protein ZO-1 in BBB endothelial cells significantly decreased in Tet2-KO mice. We also confirmed that the decreased expression of ZO-1 was positively correlated with the decreased level of 5hmC of endothelial cells in aged human brains. In cultured endothelial cells, we verified that oxidative stress significantly attenuated TET2 function, increased the methylation level of CpG islands in the promoter region of the ZO-1 gene, and inhibited ZO-1 expression. Due to the accumulation of oxidative metabolites in the process of aging, we hypothesize that inhibition of TET2 demethylase activity of endothelial cells leads to the destruction of BBB integrity, which plays a role in aging. Sasaki et al. found that isocitrate dehydrogenase 1 (IDH1) mutation produced a large amount of metabolites, such as 2-HG, which competitively inhibited α- kG dependent TET2 activity, leading to altered regulation of type IV collagen maturation and incomplete basement membrane of cerebral microvessels [27]. These lead to perinatal intracerebral hemorrhage and death in mice. Since cerebral microvascular endothelial cells are one of the critical components of BBB, our findings strengthen the correlation between the destruction of BBB and the inhibition of TET2 activity.

In a human study, BBB impairment in aged brains was also correlated with increased inflammatory markers in the cerebrospinal fluid [22]. BBB impairment might occur after the decreased expression of ZO-1, which is related to aging-induced cytokine changes [28]. It recognized that plasma proteins leak into the brain parenchyma after BBB damage. Plasma proteins, such as thrombin, plasmin, albumin, and other proteins like immunoglobin, not only cause direct damage to neurons but also activate microglia and astrocytes to aggravate inflammatory neurotoxicity [29]. However, Goodall et al. reported that the human brain staining showed BBB damage in aged brains and associated reactive astrogliosis, but no age-associated changes in microglia pathology, microvascular density, and pericyte coverage [28]. Whether compromise of the integrity of BBB is involved in decreasing the number of neurons in aged brains and the role of astrocyte and oligodendrocyte activation in variable neurological diseases still need to be further studied.

Disruption of the BBB coincides with healthy aging, but the breakdown can become further exacerbated in neurodegenerative disorders. BBB disruption, supported by the reduction of tight junction proteins, is a hallmark of a number of neurodegenerative diseases, such as AD, Parkinson’s disease (PD), amyotrophic lateral sclerosis (ALS), multiple sclerosis (MS), and Huntington’s disease (HD) [29, 30]. The disruption of the BBB in neurodegenerative disorders makes it a clear target for developing new therapeutics. If the BBB disruption could be restored, at least to some extent, it may be possible to slow down the progression of certain conditions. Emerging research on the gut-brain axis and the protection or disruption of gut microbial-derived metabolites on BBB integrity is only at the very beginning stage. To better understand the role of epigenetic modulation of the BBB integrity, especially the participation of TET enzymes whose activity could be regulated by various factors and pathological conditions, it will be interesting to incorporate and utilize these interactions to develop therapeutics aiming to either restore or protect against BBB breakdown. A good example is the administration of vitamin C, which is a potent enhancer of TET activity. It can be taken as a supplement to prevent damage to the BBB and associated CNS conditions [30–32].

## Supplementary Information

**Additional file 1: Figure S1.** The expressions of claudin-5 in mouse brains. **A**, **B** Claudin-5 expression by endothelial cells of 12- month old wild type (**A**) and KO mice (**B**). **Figure S2.** The expressions of claudin-5 in cultured endothelial cells. **A** The expression of Claudin-5 in endothelial cells treated with or without 10 μM H_2_O_2_ for 6 h and supplemented with or without 1 mM NAC for an extended 6 h. **B** The expression of Claudin-5 in endothelial cells after Tet2 was knocked down by siRNA. **Figure S3.** The expression of claudin-5 in endothelial cells of human brains. **A**, **B** The number of claudin-5 positive cells/field in the adult group (**A**) and the aged group (**B**). **C**. Semi-quantitative analysis of claudin-5 staining. Scale bars: 50 μm. All data were shown as the mean ± SEM. The p values were determined by the two-tailed t-test. Values of p < 0.05 were considered statistically significant. * denoted p < 0.05; ns, not significant.

## Data Availability

All data produced for this manuscript are available from the corresponding authors upon reasonable request.

## References

[1] Pujadas EFA (2012). Regulated noise in the epigenetic landscape of development and disease. Cell.

[2] Ramsahoye BHBD, Lyko F, Clark V, Bird AP, Jaenisch R (2000). Non-CpG methylation is prevalent in embryonic stem cells and may be mediated by DNA methyltransferase 3a. Proc Natl Acad Sci USA.

[3] Tahiliani MKK, Shen Y, Pastor WA, Bandukwala H, Brudno Y, Agarwal S, Iyer LM, Liu DR, Aravind L, Rao A (2009). Conversion of 5-methylcytosine to 5-hydroxymethylcytosine in mammalian DNA by MLL partner TET1. Science.

[4] Wu XZY (2017). TET-mediated active DNA demethylation: mechanism, function and beyond. Nat Rev Genet.

[5] Branco MR, Ficz G, Reik W (2011). Uncovering the role of 5-hydroxymethylcytosine in the epigenome. Nat Rev Genet.

[6] Blaschke K, Ebata KT, Karimi MM, Zepeda-Martínez JA, Goyal P, Mahapatra S, Tam A, Laird DJ, Hirst M, Rao A, Lorincz MC, Ramalho-Santos M (2013). Vitamin C induces Tet-dependent DNA demethylation and a blastocyst-like state in ES cells. Nature.

[7] Thienpont B, Steinbacher J, Zhao H, D'Anna F, Kuchnio A, Ploumakis A, Ghesquière B, Van Dyck L, Boeckx B, Schoonjans L, Hermans E, Amant F, Kristensen VN, Peng Koh K, Mazzone M, Coleman M, Carell T, Carmeliet P, Lambrechts D (2016). Tumour hypoxia causes DNA hypermethylation by reducing TET activity. Nature.

[8] Xu W, Yang H, Liu Y, Yang Y, Wang P, Kim SH, Ito S, Yang C, Wang P, Xiao MT, Liu LX, Jiang WQ, Liu J, Zhang JY, Wang B, Frye S, Zhang Y, Xu YH, Lei QY, Guan KL, Zhao SM, Xiong Y (2011). Oncometabolite 2-hydroxyglutarate is a competitive inhibitor of α-ketoglutarate-dependent dioxygenases. Cancer Cell.

[9] Meisel M, Hinterleitner R, Pacis A, Chen L, Earley ZM, Mayassi T, Pierre JF, Ernest JD, Galipeau HJ, Thuille N, Bouziat R, Buscarlet M, Ringus DL, Wang Y, Li Y, Dinh V, Kim SM, McDonald BD, Zurenski MA, Musch MW, Furtado GC, Lira SA, Baier G, Chang EB, Eren AM, Weber CR, Busque L, Godley LA, Verdú EF, Barreiro LB, Jabri B (2018). Microbial signals drive pre-leukaemic myeloproliferation in a Tet2-deficient host. Nature.

[10] Stamatovic SM, Keep RF, Andjelkovic AV (2008). Brain endothelial cell-cell junctions: how to "open" the blood brain barrier. Curr Neuropharmacol.

[11] Ballabh P, Braun A, Nedergaard M (2004). The blood-brain barrier: an overview: structure, regulation, and clinical implications. Neurobiol Dis.

[12] Schreibelt G, Musters RJ, Reijerkerk A, de Groot LR, van der Pol SM, Hendrikx EM, Döpp ED, Dijkstra CD, Drukarch B, de Vries HE (2006). Lipoic acid affects cellular migration into the central nervous system and stabilizes blood-brain barrier integrity. J Immunol.

[13] Gu Y, Chen J, Zhang H, Shen Z, Liu H, Lv S, Yu X, Zhang D, Ding X, Zhang X (2020). Hydrogen sulfide attenuates renal fibrosis by inducing TET-dependent DNA demethylation on Klotho promoter. FASEB J.

[14] Bao Y, Bai M, Zhu H, Yuan Y, Wang Y, Zhang Y, Wang J, Xie X, Yao X, Mao J, Fu X, Chen J, Yang Y, Lin W (2021). DNA demethylase Tet2 suppresses cisplatin-induced acute kidney injury. Cell Death Discov.

[15] Association WM (2013). World Medical Association Declaration of Helsinki: ethical principles for medical research involving human subjects. JAMA.

[16] Carrillo-Jimenez A, Deniz Ö, Niklison-Chirou MV, Ruiz R, Bezerra-Salomão K, Stratoulias V, Amouroux R, Yip PK, Vilalta A, Cheray M, Scott-Egerton AM, Rivas E, Tayara K, García-Domínguez I, Garcia-Revilla J, Fernandez-Martin JC, Espinosa-Oliva AM, Shen X, St George-Hyslop P, Brown GC, Hajkova P, Joseph B, Venero JL, Branco MR, Burguillos MA (2019). TET2 regulates the neuroinflammatory response in microglia. Cell Rep.

[17] Hashimoto H, Vertino PM, Cheng X (2010). Molecular coupling of DNA methylation and histone methylation. Epigenomics.

[18] Tahiliani M, Koh KP, Shen Y, Pastor WA, Bandukwala H, Brudno Y, Agarwal S, Iyer LM, Liu DR, Aravind L, Rao A (2009). Conversion of 5-methylcytosine to 5-hydroxymethylcytosine in mammalian DNA by MLL partner TET1. Science.

[19] Grammas P (2011). Neurovascular dysfunction, inflammation and endothelial activation: implications for the pathogenesis of Alzheimer's disease. J Neuroinflammation.

[20] Chen LL, Morcelle C, Cheng ZL, Chen X, Xu Y, Gao Y, Song J, Li Z, Smith MD, Shi M, Zhu Y, Zhou N, Cheng M, He C, Liu KY, Lu G, Zhang L, Zhang C, Zhang J, Sun Y, Qi T, Lyu Y, Ren ZZ, Tan XM, Yin J, Lan F, Liu Y, Yang H, Qian M, Duan C, Chang X, Zhou Y, Shen L, Baldwin AS, Guan KL, Xiong Y, Ye D (2022). Itaconate inhibits TET DNA dioxygenases to dampen inflammatory responses. Nat Cell Biol.

[21] Cimmino L, Dolgalev I, Wang Y, Yoshimi A, Martin GH, Wang J, Ng V, Xia B, Witkowski MT, Mitchell-Flack M, Grillo I, Bakogianni S, Ndiaye-Lobry D, Martín MT, Guillamot M, Banh RS, Xu M, Figueroa ME, Dickins RA, Abdel-Wahab O, Park CY, Tsirigos A, Neel BG, Aifantis I (2017). Restoration of TET2 function blocks aberrant self-renewal and leukemia progression. Cell.

[22] Bowman GL, Dayon L, Kirkland R, Wojcik J, Peyratout G, Severin IC, Henry H, Oikonomidi A, Migliavacca E, Bacher M, Popp J (2018). Blood-brain barrier breakdown, neuroinflammation, and cognitive decline in older adults. Alzheimers Dement.

[23] Jessop P, Toledo-Rodriguez M (2018). Hippocampal TET1 and TET2 expression and DNA hydroxymethylation are affected by physical exercise in aged mice. Front Cell Dev Biol.

[24] Stamatovic SM, Johnson AM, Keep RF, Andjelkovic AV (2016). Junctional proteins of the blood–brain barrier: New insights into function and dysfunction. Tissue barriers.

[25] Lochhead JJ, Yang J, Ronaldson PT, Davis TP (2020). Structure, function, and regulation of the blood–brain barrier tight junction in central nervous system disorders. Front Physiol.

[26] Erdő F, Denes L, de Lange E (2017). Age-associated physiological and pathological changes at the blood–brain barrier: a review. J Cereb Blood Flow Metab.

[27] Sasaki M, Knobbe CB, Itsumi M, Elia AJ, Harris IS, Chio II, Cairns RA, McCracken S, Wakeham A, Haight J, Ten AY, Snow B, Ueda T, Inoue S, Yamamoto K, Ko M, Rao A, Yen KE, Su SM, Mak TW (2012). D-2-hydroxyglutarate produced by mutant IDH1 perturbs collagen maturation and basement membrane function. Genes Dev.

[28] Goodall EF, Wang C, Simpson JE, Baker DJ, Drew DR, Heath PR, Saffrey MJ, Romero IA, Wharton SB (2018). Age-associated changes in the blood–brain barrier: comparative studies in human and mouse. Neuropathol Appl Neurobiol.

[29] Abrahamson EE, Ikonomovic MD (2020). Brain injury-induced dysfunction of the blood brain barrier as a risk for dementia. Exp Neurol.

[30] Knox EGAM, Clarke G, Cryan JF, O'Driscoll CM (2022). The blood-brain barrier in aging and neurodegeneration. Mol Psychiatry.

[31] Brabson JP, Leesang T, Mohammad S, Cimmino L (2021). Epigenetic regulation of genomic stability by vitamin C. Front Genet.

[32] Bensberg M, Rundquist O, Selimović A, Lagerwall C, Benson M, Gustafsson M, Vogt H, Lentini A, Nestor CE (2021). TET2 as a tumor suppressor and therapeutic target in T-cell acute lymphoblastic leukemia. Proc Natl Acad Sci USA.

