# Supplementary material for: ROS attenuates TET2-dependent ZO-1 epigenetic expression in cerebral vascular endothelial cells
Source: Fluids Barriers CNS. 2022 Sep 8;19:73. doi: 10.1186/s12987-022-00370-8 (PMC9461112; doi:10.1186/s12987-022-00370-8)
Supplement: Supplementary file 1 — Additional file 1: Figure S1. The expressions of claudin-5 in mouse brains. A, B Claudin-5 expression by endothelial cells of 12- month old wild type (A) and KO mice (B). Figure S2. The expressions of claudin-5 in cultured endothelial cells. A The expression of Claudin-5 in endothelial cells treated with or without 10 μM H2O2 for 6 h and supplemented with or without 1 mM NAC for an extended 6 h. B The expression of Claudin-5 in endothelial cells after Tet2 was knocked down by siRNA. Figure S3. The expression of claudin-5 in endothelial cells of human brains. A, B The number of claudin-5 positive cells/field in the adult group (A) and the aged group (B). C. Semi-quantitative analysis of claudin-5 staining. Scale bars: 50 μm. All data were shown as the mean ± SEM. The p values were determined by the two-tailed t-test. Values of p < 0.05 were considered statistically significant. * denoted p < 0.05; ns, not significant. [file 12987_2022_370_MOESM1_ESM.docx]

**Additional file 1**


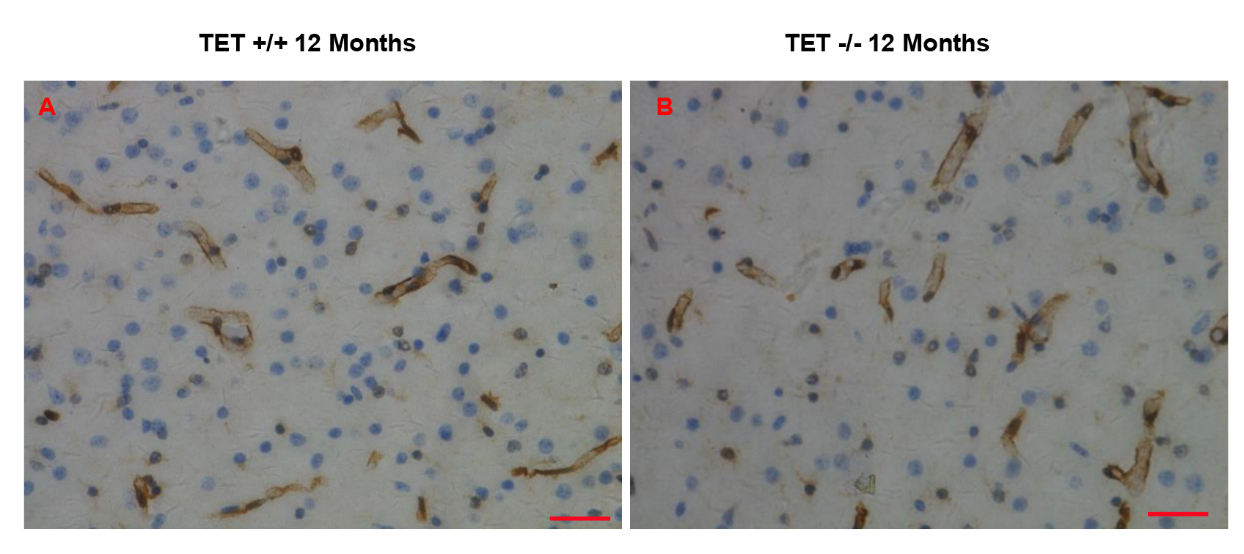


**Fig. S1.** The expressions of claudin-5 in mouse brains. **A-B** Claudin-5 expression by endothelial cells of 12- month old wild type (**A**) and KO mice (**B**).


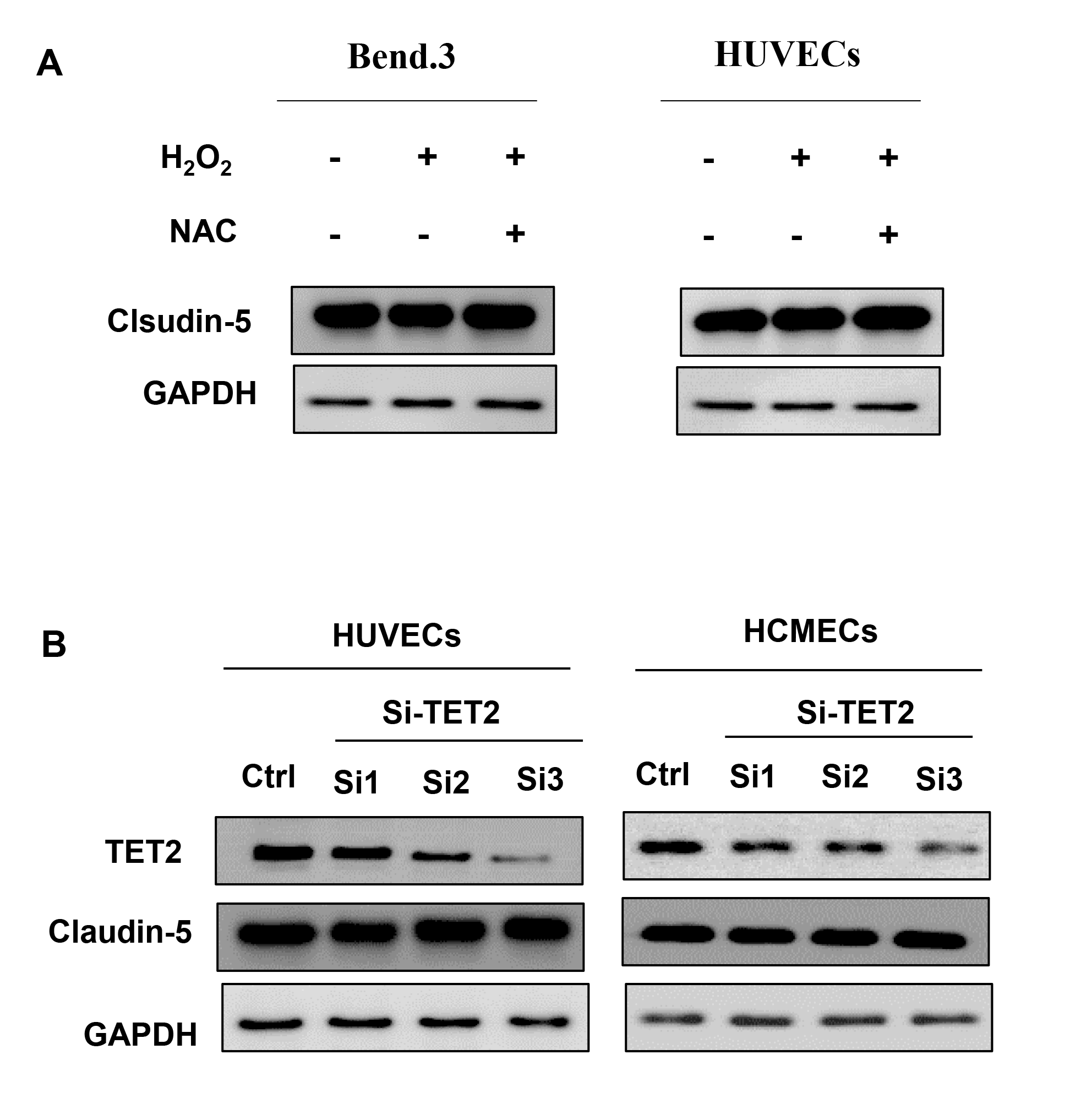


**Fig. S2.** The expressions of claudin-5 in cultured endothelial cells. **A.** The expression of Claudin-5 in endothelial cells treated with or without 10 μM H_2_O_2_ for 6 h and supplemented with or without 1 mM NAC for an extended 6 h. **B.** The expression of Claudin-5 in endothelial cells after Tet2 was knocked down by siRNA.


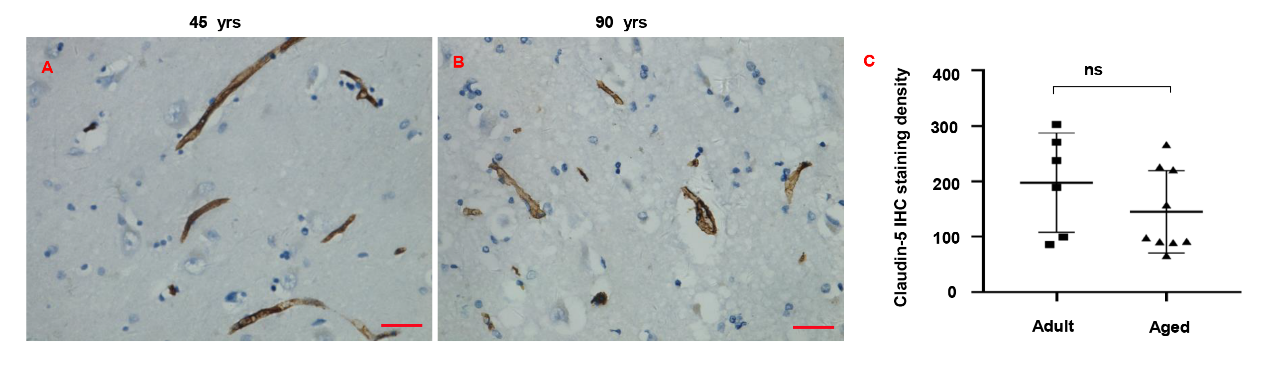


**Fig. 3S.** The expression of claudin-5 in endothelial cells of human brains. **A-B.** The number of claudin-5 positive cells/field in the adult group (**A**) and the aged group (**B**). **C**. Semi-quantitative analysis of claudin-5 staining. Scale bars: 50 μm. All data were shown as the mean ± SEM. The p values were determined by the two-tailed t-test. Values of p < 0.05 were considered statistically significant. * denoted p <0.05; ns, not significant.


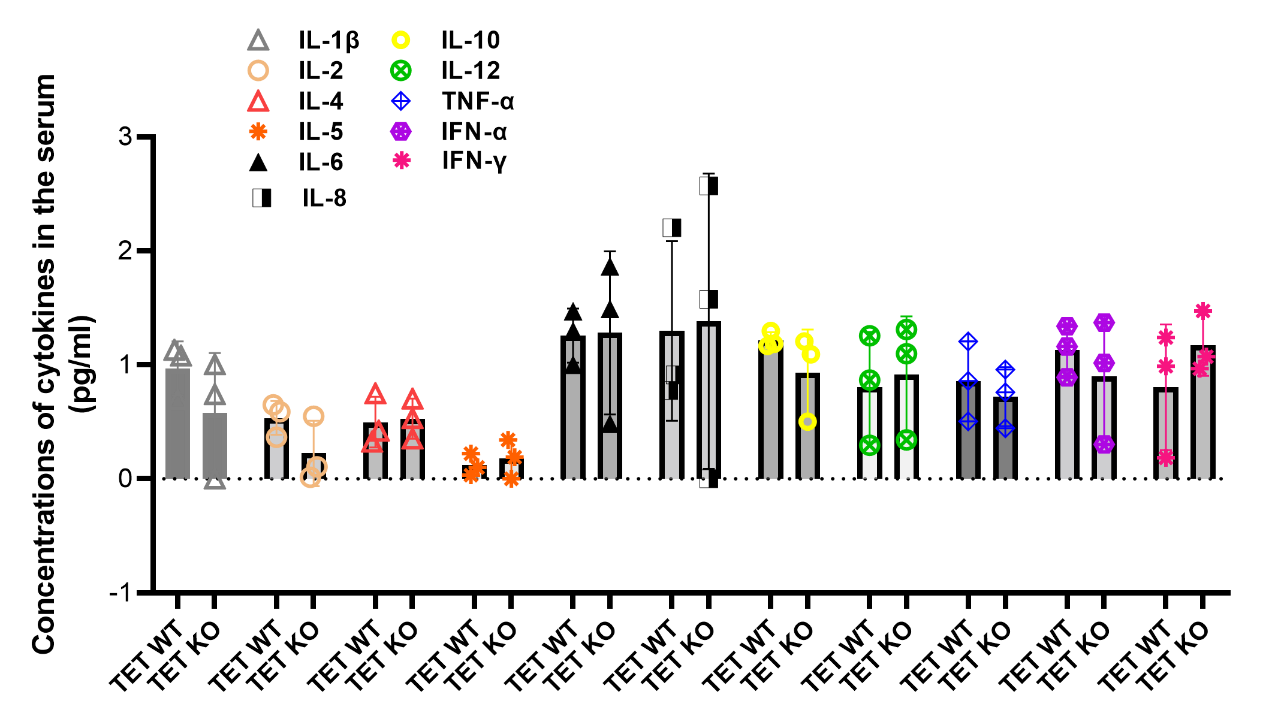


**Fig. S4.** The expression of cytokines in the serum of wild type and Tet2 KO mice. No statistical difference in levels of 11 cytokines was observed between wild-type and Tet KO mice. All data were shown as the mean ± SEM. The p values were determined by Two-way ANOVA. Values of p < 0.05 were considered statistically significant.
